# Bright-White Beetle Scales Optimise Multiple Scattering of Light

**DOI:** 10.1038/srep06075

**Published:** 2014-08-15

**Authors:** Matteo Burresi, Lorenzo Cortese, Lorenzo Pattelli, Mathias Kolle, Peter Vukusic, Diederik S. Wiersma, Ullrich Steiner, Silvia Vignolini

**Affiliations:** 1 European Laboratory for Non-linear Spectroscopy (LENS), Università di Firenze, 50019 Sesto Fiorentino (FI), Italy; 2 Istituto Nazionale di Ottica (CNR-INO), Largo Fermi 6, 50125 Firenze (FI), Italy; 3 Università di Firenze, Dipartimento di Fisica e Astronomia, 50019 Sesto Fiorentino (FI), Italy; 4 School of Engineering and Applied Sciences Harvard University 29 Oxford St., Cambridge, MA, 02138, USA and Department of Mechanical Engineering, Massachusetts Institute of Technology, 77 Massachusetts Avenue, Cambridge, MA 02139, USA; 5 Thin Film Photonics, School of Physics, Exeter University, Exeter EX4 4QL, UK; 6 Cavendish Laboratory, Department of Physics, University of Cambridge, J. J. Thomson Avenue, Cambridge CB3 0HE, U.K; 7 Department of Chemistry, University of Cambridge Lensfield Road, Cambridge CB2 1EW UK

## Abstract

Whiteness arises from diffuse and broadband reflection of light typically achieved through optical scattering in randomly structured media. In contrast to structural colour due to coherent scattering, white appearance generally requires a relatively thick system comprising randomly positioned high refractive-index scattering centres. Here, we show that the exceptionally bright white appearance of *Cyphochilus* and *Lepidiota stigma* beetles arises from a remarkably optimised anisotropy of intra-scale chitin networks, which act as a dense scattering media. Using time-resolved measurements, we show that light propagating in the scales of the beetles undergoes pronounced multiple scattering that is associated with the lowest transport mean free path reported to date for low-refractive-index systems. Our light transport investigation unveil high level of optimisation that achieves high-brightness white in a thin low-mass-per-unit-area anisotropic disordered nanostructure.

Complex photonic nanostructures in nature are synthesised in ambient conditions using a limited range of component materials. Given these limitations, an amazing range of optical strategies exists, optimised by at least 500 million years of evolution^1^,^2^,^3^. In addition to brilliant colouration for communication^4^,^5^, mating^6^ and camouflage^7^,^8^, the control of material morphologies influences thermoregulation^9^ and provides adhesive^10^ and hydrophobic properties^11^. Functional structures in insects mostly consist of chitin and melanin^12^. The assembly of these materials in various parts of the body often creates intriguing optical effects ranging from matte to iridescent colours^12^,^13^, and from black^14^ to substitutes extremely with bright white^15^,^16^,^17^. These structural colours arise from complex nanostructures such as ordered and quasi-ordered photonic crystals and random assemblies^18^,^19^,^20^.

Periodic photonic structures in nature are advantageous for many insects since a thickness of only a few micrometers is sufficient to obtain high optical reflectivities^21^. This is particularly important for insect flight, where the weight and function of optical materials must be carefully balanced. While sub-micrometer thick films are enough to generate colours by using interference effects, a bright white usually involves much thicker layers since it requires optical processes arising from multiple light scattering. Intense white reflection (above 70%) from the scales of the *Cyphochilus* and *Lepidiota stigma* beetles arises however from only 5–15 μm thick layers made of a very dense interconnected random network of chitin (volume fraction 50–60%) of refractive index *n*_c_ = 1.56^16^,^22^. Efficient broadband reflection and such dense morphology are surprising, since it is well known that high density strongly limits the scattering strength^23^. How these chitin morphologies achieved highly efficient light scattering is currently unknown.

Here, we elucidate the structural and optical optimisation of the mechanism underpinning the exceptionally bright whiteness of the scales of these two beetle species. By performing ultra-fast time-resolved measurements, we show that light propagating in the scales undergoes pronounced multiple scattering. Only the use of diffusion theory allows to retrieve the characteristic quantities describing light transport in random media, such as the transport mean free path. Moreover we find that the scales are among the most strongly-scattering low-refractive-index materials known. Such a high scattering strength is achieved by taking advantage of the anisotropic shape and orientation of the scattering elements forming the disordered network. The anisotropy of the scattering nanostructure combined with its remarkably low mass per unit area – crucial for flying insects – are evidence for optimisation by evolution.

## Results

### Structure of specimens

**Figure 1:**
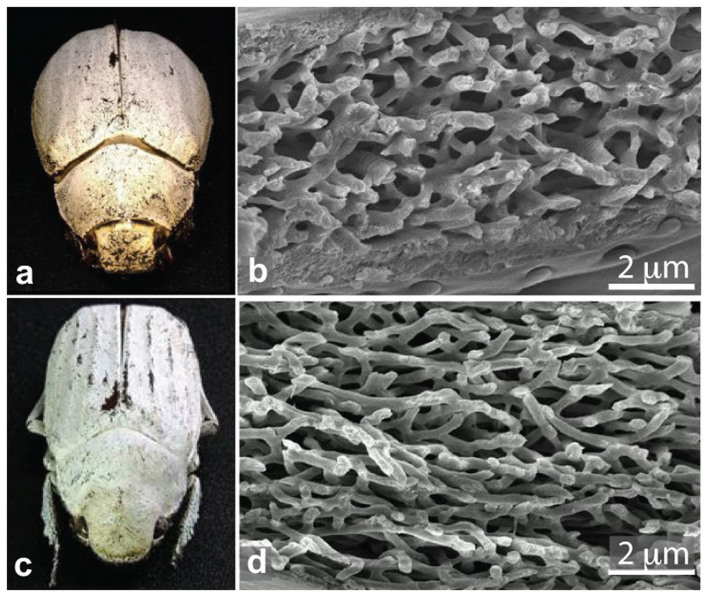
White reflection from beetle scales. (a,c), Images of *Cyphochilus* and *Lepidiota stigma* beetles, respectively. (b,d), Scanning electron micrographs (SEM) of the cross-section of the scales of the respective species.

Single scales of the *Cyphochilus* and *Lepidiota stigma* beetles (Fig. 1a,c) were investigated. The scanning electron micrographs (SEMs) in Fig. 1b,d shows cross-sections of the investigated scales and reveal their internal structure. Several scale were detached from the beetles and mounted onto glass slides for the optical measurements. After the measurements, the same scales were transferred onto SEM stubs and cross-sectioned by focused ion beam milling. The scales appear white at any angle of observation, which was caused by the scattering of light occurring in the internal random network of interconnecting rod-like filaments of chitin (Fig. 1)^22^. This is in contrast to the white pierid butterflies, in which moderate white brightness is caused by single scattering from a layer of randomly arranged granules^24^. The thickness of the beetle scales of only 8 μm and 15 μm^22^ and the relatively low refractive index of chitin raise the question whether single or multiple scattering lies at the origin of their bright whiteness, and how their scattering strength is optimized.

### The brightness

To shed light on this topic, we have measured the time-of-flight of a light pulse through single scales. These measurements separate the ‘early' light, which undergoes only few scattering events, from the ‘late' light, characteristic for multiple scattering^25^,^26^. An ultra-fast time-resolved experiment using an optical gating technique^27^ with ultra-short pulses (≈100 fs) was employed. The variation of the temporal delay between a probe pulse at 810 nm and a gate pulse at 1550 nm impinging on a nonlinear crystal allows the reconstruction of their temporal cross-correlation by detecting the sum-frequency signal. The length of the pulses was about 30 μm, which is longer than the thickness of the scales. To accurately assess the photon lifetime, a significant decay of the optical signal has to be measured in order to identify a clear exponential signal variation, which requires a very large dynamic range of the detector. In our case, a photomultiplier and a photon counter provided a detection dynamic range up to 10^7^. This optical setup has been carefully optimized for the investigation of thin disordered materials (see Methods and Supplementary Information)^28^,^29^.

**Figure 2:**
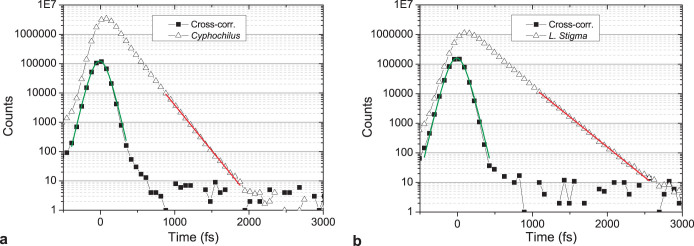
Time-resolved measurements of the scales. (a,b) Time-of-flight of light transmitted through the scales of *Cyphochilus* and *L. stigma*, respectively (open triangles). The reference measurement (cross-correlation) of the probe pulse is shown as black squares. The fit of the probe pulse (green line) yields a pulse duration, in semilog scale of 130 fs. In contrast, the pulse transmitted through the scales exhibits an exponential tail over three orders of magnitude in intensity. The exponential fit (red line) yields lifetimes of *τ* ≈ 140 fs and *τ* ≈ 210 fs for (a,b), respectively.

As discernible in the SEMs of Fig. 1b,d, and the Supplementary Information, the thickness and curvature vary along the scales. The time-resolved experiments were performed by exciting a spot with approximately 2 μm diameter on the central part of the scale. Figure 2 shows typical time-resolved transmission measurements through *Cyphochilus* and *L. stigma* scales on a semilogarithmic scale. The data were obtained by averaging over several measurements probing an area of approximately 10 × 10 μm^2^. The shape of the probe pulse can be fitted with the convolution of two squared hyperbolic secants, which yields a pulse duration of about 130 fs. In contrast to the reference signal, a clear delay and deformation of the pulse is induced by its interaction with the samples.

This is a clear evidence of a multiple light scattering mechanism in these ultra-thin beetle scales. Firstly, single light scattering would be characterised by a pulse-peak delay of only few fs (comparable with the ballistic time), whereas the measured delays are approximately 80 fs and 140 fs. Secondly, in the case of single scattering, the pulse shape is nearly unmodified, whereas exponential tails with stable slopes over 3 orders of magnitude in intensity are observed in Fig. 2. This is a clear fingerprint that light has been diffusively captured in the scales and reemerges at late times. By fitting the exponential tail, the average residing time of light in the scales, the so-called photon lifetime, was determined. Values of *τ* ≈ 140 fs and *τ* ≈ 210 fs are found for *Cyphochilus* and *L. stigma* scales, respectively.

Based on this evidence for multiple light scattering in the scales, it is reasonable to describe light propagation in the random networks by applying diffusion theory^30^, which provides profound physical insights in the transport properties of disordered media and it has been proven to work well even for thin materials^28^,^31^,^32^. Diffusion theory relates the measured decay time *τ* to the diffusion coefficient *D* and the transport mean free path *ℓ*_t_, which is the de-correlation length, i.e. the average distance after which light loses memory of its propagation direction, 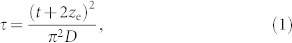
where *t* is the thickness of the disordered material, *D* = *v*_e_*ℓ*_t_/3, *v*_e_ is the transport velocity, *z*_e_ = (2*ℓ*_t_/3)(1 + *R*)/(1 − *R*) is the so-called extrapolation length, and *R* is the integrated reflection coefficient due to the contrast between the surrounding air and the effective refractive index *n*_e_ of the scale interface^33^ (see Methods). The derivation of the effective refractive index of a disordered medium is always a delicate matter. However, as shown in previous works^34^,^35^,^36^,^37^, in case of dense scattering media, *n*_e_ can be approximately calculated using the Maxwell Garnett mixing rule as a function of the cuticle volume fraction *f* (see below) and thus also *R* is *f*-dependent.

The use of diffusion theory for the beetle scales needs some further discussion due to the scale network morphology. Indeed, the diffusion equation for light is retrieved in the independent scattering approximation^30^, which is easily satisfied when the system is a diluted assembly of distinct point-like scatters with a well-defined scattering cross-section. In the case of a continuous tubular, dense random network, however, it is not possible to clearly define individual scatterers and their scattering properties are influenced by the presence of the nearest neighbours. Nevertheless, we can still employ diffusion theory to investigate the optical properties of the scales by describing them as an ‘effective diffusive media' characterized by an effective transport mean free path *ℓ*_t_ which can be inserted in equation 1. Also, the finite size of the scatterers requires a modification of the transport velocity *v*_e_, which can be approximated by *v*_e_ = *c*/*n*_e_, where *c* is the speed of light^38^. This approximation has been shown to be accurate for *f* ≥ 0.50 and/or for scatterer sizes smaller than wavelength of light in the material^34^. Finally, equation 1 accurately describes the propagation of light only in optically thick (*OT*) materials, i.e. *OT* = *t*/*ℓ*_t_ ≥ 8^25^,^26^. As *OT* decreases, the accuracy of equation 1 increasingly diminishes and this has to be taken into account in our analysis.

**Figure 3:**
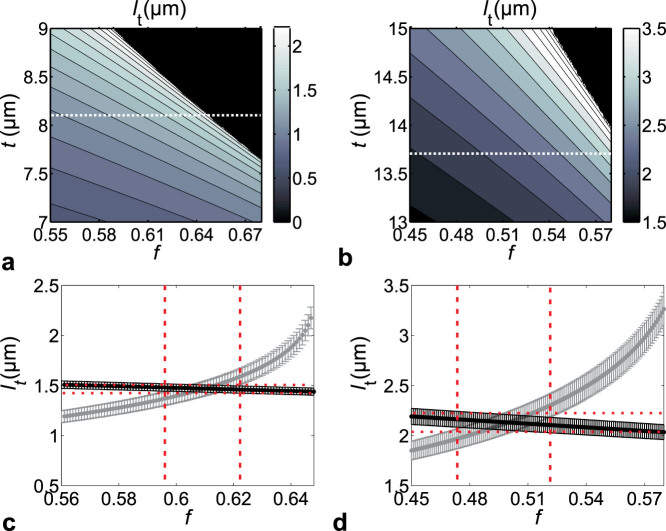
Transport mean free path in the scales. (a,b), Transport mean free path *ℓ*_t_ as a function of filling fraction *f* of the chitin random network and scale thickness *t* calculated by equation 1 using measured *τ*-values, for *Cyphochilus* and *L. stigma*, respectively. The black areas correspond to unphysical solutions. *ℓ*_t_ is smaller than 5 μm in the relevant *f* and *t* range. (c,d), Corresponding variation of *ℓ*_t_ with *f* for fixed *t* (*t* = 8.1 μm and *t* = 13.7 μm for (c,d), respectively). The grey and black symbols correspond to the predictions of equation 1 and 2, respectively. The crossing points provide estimates of *ℓ*_t_ and *f*, delimited by the confidence range indicated by dashed lines.

The thickness of the scales is readily measured from SEM images (see Supplementary Information), yielding *t* = (8.1 ± 0.2) μm and *t* = (13.7 ± 0.5) μm for *Cyphochilus* and *L. stigma*, respectively. A direct determination of the filling fraction is however difficult^22^. Figure 3a,b shows the prediction of *ℓ*_t_ from equation 1 as a function of *t* and *f* for the two beetle species. Clearly, *ℓ*_t_ varies between 1 to 2.5 μm and 2 to 4.5 μm for *Cyphochilus* and *L. stigma*, respectively. Therefore, for all physically relevant values of *t* and *f*, *ℓ*_t_ is predicted to lie below 5 μm in both scales, which is a remarkably short transport mean free path for such a low-refractive-index material.

### Static experiments

An independent way to estimate *ℓ*_t_ as a function of *f* is the measurement of the total transmission of light, *T*_tot_, using the same effective diffusive medium approach as described above. Given the small optical thickness of the samples, we initially considered a formula which takes into account both the multiple scattering and the ballistic contribution to the transmission^32^ as 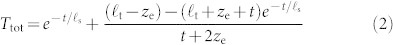
where *ℓ*_s_ = *ℓ*_t_(1 − *g*) is the scattering mean free path and *g* is the so-called anisotropic factor of the scatterers^39^. Although *g* is not known for these complex structures, it lies between 0 and 1 and thus *ℓ*_s_ is always smaller than *ℓ*_t_. From the values of *ℓ*_t_ in Fig. 3a,b, the ballistic component in equation 2 turns out to be negligible and thus the well-known Ohm's law for light can be employed: 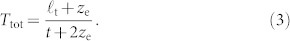
The total transmission *T*_tot_ was measured using an integrating sphere which collects all light emerging from a single scale. This steady state experiment was performed using the same probing conditions as in the time-resolved measurements (see Methods), resulting in *T*_tot_ = 0.3 ± 0.01 and *T*_tot_ = 0.26 ± 0.01 at 810 nm for *Cyphochilus* and *L. stigma*, respectively. *ℓ*_t_ as a function of *f* was extracted by solving equation 3, shown in Fig. 3c,d (black symbols). The error bars represent the range of confidence of *ℓ*_t_, which is not only affected by the instrumental accuracy but also by the difficulty in determining the boundaries of the disordered network. The grey symbols in Fig. 3c,d correspond to the values of the white dashed lines in Fig. 3a,b. From previous studies on transport in disordered thin films^28^, we expect an accuracy of about 5% when determining *ℓ*_t_ in the beetle scales from equation 1. The intersections of the independent data sets provide estimates for the network volume fractions of *f* = 0.61 ± 0.02 and *f* = 0.50 ± 0.03 for *Cyphochilus* and *L. stigma*, respectively. These values are in good agreement with the previously reported values of *f* = 0.68 ± 0.07 and *f* = 0.48 ± 0.03 for *Cyphochilus* and *L. stigma*, respectively^22^. Following this analysis, we retrieve all the relevant parameters characterising the multiple scattering of light, as shown in Table 1.

The striking scattering strength of these biological materials is revealed when comparing them to other white samples in Table 1 with similar refractive index. Paper consists of an anisotropic network of fibres (size of hundreds of microns) with a refractive index (≈1.48) that is only little lower than chitin and *f* = 0.5. The syringe filter is made of an isotropic network of fibres (size of hundreds of nanometres) also made of cellulose with *f* = 0.3. Despite the structural similarities both beetle scales have a significantly lower transport mean free path. Photonic Glasses, on the other hand, are made from microscopic polystyrene spheres (refractive index 1.5) with *f* = 0.55. These materials have been engineered to resonantly scatter light^35^,^40^ and yet exhibit a scattering strength weaker than both types of scales. To our knowledge, the beetle scales exhibit the lowest transport mean free path length and diffusion constants reported for a low-refractive-index material (*n* ≈ 1.5). These values are even more intriguing when considering the high filling fraction of the scales. It is well known that increasing *f* does not lead to a linear increase of the scattering strength of a disordered material^23^. The scattering efficiency exhibits a maximum, generally around *f* = 0.2, and decreases continously as *f* increases beyond that. This effect is called ‘optical crowding', that is, the reduction of the scattering of individual scatterers due to the near presence of others. For comparison, let us consider a random assembly of spherical particles with *n* = 1.5 and *f* = 0.2. Employing Mie theory in the independent scattering approximation and taking into account the short-range correlations caused by sphere packing^41^, we find 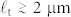 for any particle size (see Supplementary Information). It is crucial to note that this approximation *underestimates* *ℓ*_t_, since it does not consider optical crowding or near-field interactions between scatterers which dominates in real systems with high *f*-values^23^,^42^,^43^. In this context, the values of 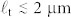 of the *Cyphochilus* and *L. stigma* scales is surprising, because their filling fraction is significantly higher than 0.2. This effect can be understood considering the scatterers shape and the high filling fraction of the network. The ensembles of strongly anisotropic scatterers can be packed with high *f*-values introducing a certain degree of anisotropy in the systems, since the scatterers have to align on average with a planar orientation^44^. Indeed, in the SEM cross-sections of the scales (Fig. 1 and Supplementary Video) the elongated chitin elements that constitute the network are mainly oriented parallel to the scale surface. Moreover, the constraint to have a continuous random network (and not isolated scattering elements) imposes the scattering elements to be connected and reduces the contact surfaces between much of the surface of the scatterers (i.e. only at the extremities), even for very high *f*-values. Combining these two properties of the network, the beetles succeed in increasing the scattering strength along the direction normal to the scale surface, while concurrently decreasing the effect of optical crowding. Such effect can be understood considering a decrease of the scattering strength for light propagating parallel to the scale. The highly debated effect of this anisotropy on light transport^45^,^46^,^47^ will be further investigated in future works. It is remarkable to note, however, that the brightness of the scales, as well as the decay time *τ*, is only dictated by the scattering strength along the direction normal to the scale, as discussed in the Supplementary Information.

The high brightness of the two beetles scales is achieved in both cases by employing a relatively high material density. Interestingly, the two beetle species have optimised the parameters of the scattering layer in a different way to achieve a similar degree of brightness, expressed as the optical thickness *OT* ≈ 6. In both cases, this reflects a careful trade-off between brightness and mass density per area. A layer containing too few scatterers lowers the optical thickness, enabling substantial light transmission and thereby low reflectivity. Interestingly, at the very *OT*-value of the scales, the ballistic light vanishes (less than 0.1%, see equation 2) and thus practically all light is scattered by the scale. Too many scatterers, on the other hand, increase the mass per unit area of the white layer. This is emphasised by comparing the materials in Table 1, in terms of their normalised optical thickness, *OT*_n_ = *OT*/*γ*, where *γ* is the specific weight per unit area (see Methods). The beetle scales clearly outperform the other materials based on biological fibrous networks and are even more efficient at scattering light than the artificially engineered Photonic Glasses. Although polystyrene has a higher refractive index and a lower density than chitin, the *OT*_n_-value for this man-made material is lower than in the case of the two beetle scales.

### The whiteness

**Figure 4:**
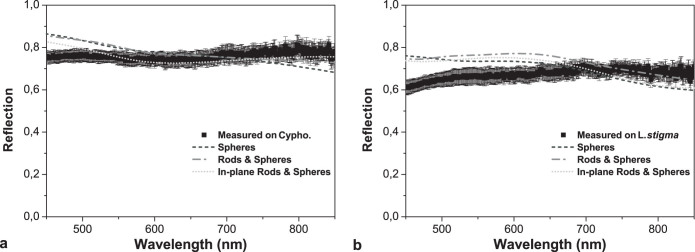
Reflection spectra of the white scales. (a–b) The measured spectra for *Cyphochilus* and *L. stigma* scales, respectively, reveal the whiteness of a single scale which extends to the near-infrared. The dashed lines are predictions from equation 2 (*R* = 1 − *T*). The mean free path used in equation 2 is calculated by modelling the chitin continuous random network with a distribution of particle havening different shape and orientations: only spheres, spheres and rods randomly oriented in space, and spheres and rods with a random in-plane orientation (no vertically oriented rods are present).

A further characteristic property of the two beetles is the broadband whiteness which extends into the near-infrared (Fig. 4). The spectral response of single scales in reflection was measured using an optical microscope in reflection configuration. White light from a halogen source was focused onto the scale using a 50× objective. By keeping the field aperture closed, the numerical aperture of the illumination was NA = 0.55. The reflected intensity *R* was collected with the same objective in confocal configuration using the full numerical aperture of the objective of NA = 0.8. In this way it is possible to obtain the reflected intensity emerging from an area of about 10 μm diameter, integrated over all angles within the objective's NA. Figure 4 shows the measured reflection spectra of scales from both beetles, exhibiting a nearly constant response in the 450–850 nm wavelength range, suggesting that the scale transport properties do not significantly vary for different colours. Note that the reflection for *L. stigma* is lower at short wavelengths due to the presence of melanin (see Supplementary Information). On one hand, this is in contrast with the reflection properties of diluted (polydispersed) systems for which a linear spectral dependence is expected^48^. On the other hand, from the observation of a featureless reflection spectra we infer the absence of relevant spatial correlations in the distribution of scatterers inside the scale. This would not be possible, for instance, in a dense disordered assembly of spheres, since their physical extent induces a spatial order and thus a colouration in reflection, as observed in certain birds^49^. In contrast, the use of anisotropic scatterers allows to realise highly dense systems without introducing relevant spatial correlations. Since in absence of correlations the spectral response is dictated only by the refractive index and shape of the scatterers, we have developed a simple model to show that the spectrum of the reflectance is unaltered by the anisotropy of the scatterers.

The morphology of the tubular network makes the modelling of the reflectance more complex compared to an assembly of point-like scatterers. The random cuticle network can be approximated in terms of two structural elements: (1) an ensemble of dielectric rods which are randomly distributed with random orientations, and (2) a distribution of rod-junctions. We have calculated the transport cross-section of two types of scatterers, namely spheres and cylinders, to take into account the anisotropy of the scale scatterers. The former can be calculated with Mie theory^39^, approximating junctions of equal volume (diameters of 300 nm and 430 nm for *Cyphochilus* and *L. stigma*, respectively). The latter is attained by employing the Waterman T-matrix method^50^. We have used the algorithm based on the Mishchenko's public-domain codes^51^, released by Xu and Gustafson^52^. It allows the computation of the scattering matrix and thus the transport cross-section *σ*_trod_ of variously shaped scattering objects by numerically solving the Mie problem. Rod diameters of *d* = (244 ± 51) nm and *d* = (348 ± 77) nm for *Cyphochilus* and *L. stigma*, respectively, were used^22^. The rod-length between junctions intersections was approximated from SEMs, yielding an average of 800 nm and 900 nm for *Cyphochilus* and *L. stigma*, respectively. The junction volume was calculated considering the intersection of two orthogonal cylinders. In the independent scattering approximation *ℓ*_t_ = (*ρσ*_t_)^−1^, where *σ* is the transport cross-section and *ρ* is the density of the scatterers. We have calculated the reflection from various systems made of different particles with refractive index 1.56 using equation 2 (*R* = 1 − *T*, assuming negligible absorption), namely a system of spheres (same volume of the junctions), spheres and rods with random orientations and, finally, spheres and rods with an *in-plane* random orientations (rods oriented along the direction of the impinging light are not present). The density *ρ* has been chosen so to have the reflectivity *R* comparable to what has been measured in the scales, regardless of the actual physical size of the particles. For the systems of rods and spheres we have calculated the average transport mean free path as 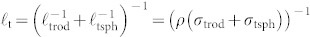, where *ℓ*_trod_ and *ℓ*_tsph_ are the transport mean free paths of the rod and sphere assemblies, respectively. The results of these calculations are shown in Fig. 4. Note that the comparison in Fig. 4 is relevant only for the spectral distribution and not for the quantitative value of the reflectance. This is due to two main reasons: (i) only the reflected light within the collection cone of the numerical aperture of objective is detected; (ii) the independent scattering approximation from which equation 2 is derived does not accurately model light transport in high-filling fraction systems. Regardless of the shape or the orientation of the anisotropic scatters the spectral response is featureless for a very broad range of wavelengths. Interestingly, the calculations shows a certain variation for *R* at short wavelengths which is not present in the measurements on the scales and thus the scales are ‘more white' than our model predicts. This suggests a certain structural optimisation since the spectral range of this whiteness exceeds what it is expected for monodisperse scattering objects.

In conclusion, we presented a detailed study of multiple scattering in single scales of *Cyphochilus* and *L. stigma* beetles. Compared to other white materials, the white reflection from these scales is produced by multiple light scattering and has clearly been optimised by natural selection to combine a low mass per unit area with bright white reflectivity. For the former, chitin is a suitable material because it combines transparency and a reasonable refractive index contrast (for biological materials) with a low mass density and a small overall layer thickness. To achieve this, the white beetles realized a peculiar anisotropic disordered medium made of a highly dense network of anisotropic scatterers. This indicates optimisation by evolution in the two white beetle species.

## Methods

### Experiments. Time-resolved measurements

Single scales were removed by scraping the beetles, placed on microscope glass slides where they are immobilised by electrostatic forces. Time-resolved and total transmission experiments were performed using a probe pulse laser at a wavelength of 810 nm, focused with an aspheric lens to a spot with diameter of about 2 μm. At this wavelength, light absorption in melanin, that is contained particularly in the *L. stigma* network (see Supporting Information), is negligible and possible optical resonant effects of the scatterers are small. The position of the illumination spot with respect to the centre of the scales was monitored by a CCD camera during the experiments. Time-resolved measurements were performed by overlapping the probe pulse with a gate pulse at 1550 nm from a parametric oscillator on a β-Barium Borate (BBO) crystal in time, space and in reciprocal space. Electromagnetic radiation with a frequency equal to the sum of the carrier frequencies of the two pulses (532 nm, green) was detected by a photomultiplier. By varying the temporal delay between the two pulses and measuring the intensity of the green light, the cross-correlation of the two pulses was reconstructed.

### Integrating sphere measurements

A laser with a wavelength of 810 nm was focused with an aspheric lens to a 2 μm-wide spot onto a scale that was fixed at the entrance of the port of an Integrating sphere. The total transmission was measured using a large area silicon photodiode and a lock-in amplifier.

### Calculation of specific weights per unit area *γ*

The specific weights per unit area is given by *γ* = *f ρt*, where *ρ* is the density. For paper and syringe filter the density of cellulose is *ρ* = 1.5 g cm^−3^, *f* = 0.5 and *f* = 0.3 and the thicknesses were approximately 113 μm and 166 μm, respectively. For a Photonic Glass made of polystyrene spheres *ρ* = 1.05 g cm^−3^, *f* = 0.55 and *t* = 50 μm. For the scales the density of chitin is *ρ* = 1.425 g cm^−3^.

### Theory

The calculation of the extrapolation length *z*_e_ in equation 1 is crucial for this study. It is well known that *z*_e_ is affected by the internal reflection *R* and can be expressed as *z*_e_ = (2*ℓ*_t_/3) *A*, where *A* = (1 + *R*)/(1 − *R*). Although the analytical expression is rather complex, 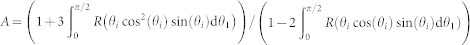, where *θ*_i_ is the angle of incidence, it as been shown^33^ that *A* can be approximated by the polynomial *A* = 504.332889 − 2641.00214*n* + 5923.699064*n*^2^ − 7376.355814*n*^3^ + 5507.53041*n*^4^ − 2463.357945*n*^5^ + 610.956547*n*^6^ − 64.8047*n*^7^, where 1 < *n* = *n*_2_/*n*_1_ < 1.6, with *n*_1_, *n*_2_ the refractive indices outside and inside the scattering medium, respectively. Using an effective medium approach, *n*_2_ = *n*_e_ where *n*_e_ was calculated with the Maxwell Garnett mixing rule: 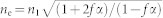 where 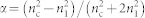.

## Author Contributions

M.B. and S.V. conceived and carried out the experiments. L.C. carried out the numerical simulations. P.V. provided the samples. M.K. performed the SEM movie in the supplementary information. All authors discussed the results and contributed to the writing of the paper.

## Supplementary Material

Supplementary Information Real time slicing of a scale

Supplementary Information SUPPLEMENTARY INFORMATION

## Figures and Tables

**Table 1:** Transport properties of the scales compared with reference samples. The errors shown on *ℓ_t_* for both scales have been estimated by the intersections of the errorbars in Fig. 3c,d

	*ℓ*_t_ (μm)	*D* (m^2^s^−1^)	*OT*	*OT*_n_ (10^3^ g^−1^cm^2^)
*Cyphochilus*	1.47 ± 0.07	112 ± 6	5.5	7.8
*L. stigma*	2.1 ± 0.1	167 ± 9	6.5	6.7
Paper	13 ± 0.65	1100 ± 55	8.7	1.0
Syringe filter	6 ± 0.3	480 ± 24	27.6	3.7
Photonic Glass	2.9	190	19	5.9
