# Supplementary material for: Bright-White Beetle Scales Optimise Multiple Scattering of Light
Source: Sci Rep. 2014 Aug 15;4:6075. doi: 10.1038/srep06075 (PMC4133710; doi:10.1038/srep06075)
Supplement: Supplementary Information [file srep06075-s2.pdf]

Supplementary Information:
Bright-White Beetle Scales Optimise Multiple Scattering of Light

Matteo Burresi[1,2], Lorenzo Cortese[1,3], Lorenzo Pattelli[1,3] Mathias Kolle[4], Peter Vukusic[5], Diederik S. Wiersma[1,2,3], Ullrich Steiner[6] & Silvia Vignolini[6]

[1] *European Laboratory for Non-linear Spectroscopy (LENS), Università di Firenze, 50019 Sesto Fiorentino (FI), Italy.*

[2] *Istituto Nazionale di Ottica (CNR-INO), Largo Fermi 6, 50125 Firenze (FI), Italy.*

[3] *Università di Firenze, Dipartimento di Fisica e Astronomia, 50019 Sesto Fiorentino (FI), Italy.*

[4] *School of Engineering and Applied Sciences and Kavli Institute for Bionano Science and Technology Harvard University 29 Oxford St., Cambridge, MA, 02138, USA.*

[5] *Thin Film Photonics, School of Physics, Exeter University, Exeter EX4 4QL, UK.*

[6] *Cavendish Laboratory, Department of Physics, University of Cambridge, J. J. Thomson Avenue, Cambridge CB3 0HE, U.K.*

1 The effect of melanin.

In order to evaluate the presence of melanin in the scales of *Cyphochilus* and *L. stigma* we performed transmission measurements on scales infiltrated with an index-matching oil. In Fig. S1a two scales from *L. stigma* (immersed in a index matching oil with $n = 1.56$) are imaged using an optical microscope in transmission configuration with a low numerical aperture objective ($\mathrm{NA} = 0.15$). The scale on the left is cut on one side and it is almost transparent due to the index matching, while the one on the right appears dark since the direct transmission is much lower due to scattering of the light outside the objective collection cone. In Fig. S1b a scale from *L. stigma* is shown, also in this case the scale has been cut to allow the index matching oil to infiltrate inside. Now a yellowish colour is more evident in different parts of the scale. This colour is the results of the presence of some melanin in the scale. The presence of melanin can be observed also if we study the transmitted light form a single scale index matched using the oil. While the transmission is flat and almost equal to 1 for all the wavelength in the case of the *Cyphochilus* beetle in all the scales considered, for the case of *L. stigma* we observe more significative absorption below 500 nm. The presence of such absorption in *L. stigma* is responsible of its more yellowish appearance when compared to the *Cyphochilus*.

2 Geometry of the investigated scales.

After all the optical experiments have been performed, the same investigated scales have been cut by focused ion beam milling and examined with a scanning electron microscope to measure their

thickness. This is a crucial step in the investigation since the dimension of the scales can vary significantly not only from animal to animal of the same species, but also from different collecting areas of the same animal. Indeed diffusion theory is extremely sensitive to the thickness t of the system and thus this parameter has to be investigated carefully. As one can see from the images in Fig. S2, the shape of the cross section of a single scale changes considerably as a function of position and thus the optically probed area is only the central part, where we estimate the thickness. Thickness and filling fraction of the structures have also been investigated using a movie composed by different SEM images of the scale taken after sequentially sectioning the scale with the FIB.

3 Transport mean free path of a particle system with $f = 0.2$.

As discussed in the main body of the manuscript, we compared the measured ℓ_t of the scales with what can be predicted by theory for a particle system with $f = 0.2$. In the independent scattering approximation the scattering mean free path ℓ_s is calculated as[1]:

$$\ell_\mathrm{s} = \left(\rho \int_0^\pi \frac{\mathrm{d}\sigma_\mathrm{s}}{\mathrm{d}\theta} \sin\theta \, \mathrm{d}\theta \right)^{-1}, \tag{1}$$

where ρ is the density of scatterers and $\mathrm{d}\sigma_\mathrm{s}/\mathrm{d}\theta$ is the differential scattering cross-section, whereas the transport mean free path ℓ_t is:

$$\ell_\mathrm{t} = \left(\rho \int_0^\pi \frac{\mathrm{d}\sigma_\mathrm{s}}{\mathrm{d}\theta} (1 - \cos\theta) \sin\theta \, \mathrm{d}\theta \right)^{-1}. \tag{2}$$

Due to the moderate but not negligible filling fraction of the particles, the light scattering becomes dependent. Short-range correlation given by their physical size, can be taken into account to calculate the transport mean free path by modelling the system as a hard-sphere fluid and applying

the Percus-Yevick equation to calculate the structure factor $S(q)$ of the system[2]. We can define a modified differential scattering cross-section[3]:

$$\frac{\mathrm{d}\sigma_s^\star}{\mathrm{d}\theta} = \frac{\mathrm{d}\sigma_\mathrm{s}}{\mathrm{d}\theta} S(q). \tag{3}$$

which can be used in equation 2 instead of $\mathrm{d}\sigma_\mathrm{s}/\mathrm{d}\theta$. In Fig. S3 is shown ℓ_t obtained by using equation 2 and 3 as a function of the particle diameter. Clearly, ℓ_t is always larger than 2 µm. Note that this calculation does not take into account the 'optical crowding' effect, for which, as far as we know, an analytical theory does not exist and which is expected to make ℓ_t even longer.

4 Time resolved optical setup for light transport investigation.

The optical gating setup employed in this investigation is schematically represented in Fig. 4. The probe beam (red), conveniently delayed by a translating stage, is focused on the entrance surface of the sample S by an aspheric lens (L2, working distance 2 mm). Particular care has been devoted to image the exit surface of the sample on the BBO nonlinear crystal by a collecting lens L3 ($f = 100$ mm). This ensures that the time-resolved data refers to light emerging from the exit surface. For this purpose a CCD camera and a flippable mirror M2 are used to monitor the alignment. The gate beam (grey) is mildly focused by the L1 lens ($f = 300\,\mathrm{mm}$) on the crystal to obtain an efficient up-conversion, whereas a dichroic mirror M1 allows the spatial superposition of the two beams. The up-converted signal is collected by the L4 lens and measured in a photon-counting regime by a photo multiplier tube (PMT). A box has been constructed to screen the PMT from environmental background signal. Varying the delay between the two laser pulses by moving the translation stage of the probe beam, we reconstruct the whole temporal evolution of the

transmitted light (see inset in Fig. S4). The fine details of the setup and the delicate up-conversion process of the diffused light will be described in a future publication.

5 Anisotropic transport and brightness of the scale.

In a structurally anisotropic disordered slab the well-known diffusion equation must be rewritten in a tensorial form which leads, under the assumption of negligible absorption and a source term equal to a point source positioned one transport mean free path away from the entering surface, to the following expression for the total transmission of the slab[4]:

$$T(x,y,t) = \frac{e^{-\frac{x^2}{4D_{xx}t}} e^{-\frac{y^2}{4D_{yy}t}}}{2(4\pi)^{3/2}\sqrt{D_{xx}D_{yy}D_{zz}}t^{5/2}} \times$$

$$\times \sum_{m=-\infty}^{\infty} \Big[z_{1,m} \exp\big(-\tfrac{z_{1,m}^2}{4D_{zz}t}\big) - z_{2,m} \exp\big(-\tfrac{z_{2,m}^2}{4D_{zz}t}\big)\Big] \quad (4)$$

where

$$z_{1,m} = L(1-2m) - 4mz_{\mathrm{e}} - \ell_{\mathrm{t}z} \quad (5)$$

$$z_{2,m} = L(1-2m) - (4m-2)z_{\mathrm{e}} + \ell_{\mathrm{t}z}, \quad (6)$$

being z_{e} the extrapolation length along z, L the thickness of the slab and t is time.

If we directly compare equation 4 with its isotropic counterpart

$$T(\rho,t) = \frac{e^{-\frac{\rho^2}{4Dt}}}{2(4\pi D)^{3/2}t^{5/2}} \sum_{m=-\infty}^{\infty} \Big[z_{1,m} \exp\big(-\tfrac{z_{1,m}^2}{4Dt}\big) - z_{2,m} \exp\big(-\tfrac{z_{2,m}^2}{4Dt}\big)\Big] \quad (7)$$

it can be noticed that the only dependence on the in-plane diffusion coefficients D_{xx} and D_{yy} is to be found in the prefactor and thus it does not influence the exponential decay of the curve at late times. The decay rate turns out to be $\tau = (L+2z_e)^2/(\pi^2 D_z)$.

The total transmission or reflection from such a slab is obtained upon spatial integration of equation 7 and 4 for the isotropic and anisotropic case, respectively. For the latter the dependence from the in-plane diffusion constants is analytically removed since $\int_{-\infty}^{\infty} \exp(-\frac{x^2}{4Dt})\,\mathrm{d}x = \sqrt{4\pi Dt}$ and we find

$$T(t) = \iint_{-\infty}^{\infty} T(x,y,t)\,\mathrm{d}x\,\mathrm{d}y = \frac{1}{2t\sqrt{4\pi D_{zz}t}} \sum_{m=-\infty}^{\infty} \Big[\cdots\Big] \tag{8}$$

which is equal to the isotropic solution as long as we consider an isotropic system with $D = D_{zz}$. The same considerations hold for any space-integrated reflection measurement as well. In other words, the amount of light diffusely transmitted or reflected in a slab geometry depends *solely* on the transport properties along the thickness direction. From a yet different perspective, we can say that any space-integrated (both static and dynamic) transmission measurement can bring no information about the in-plane transport coefficients, since it is completely unaffected by their values[5].

1. van de Hulst, H. C. *Light scattering by small particles* (Dover Publications, 1981).

2. Rojas-Ochoa, L. F., Mendez-Alcaraz, J. M., Sáenz, J. J., Schurtenberger, P. & Scheffold, F. Photonic properties of strongly correlated colloidal liquids. *Physical Review Letters* **93**, 073903 (2004).

3. Conley, G. M., Burresi, M., Pratesi, F., Vynck, K. & Wiersma, D. S. Light transport and localization in two-dimensional correlated disorder. *Physical Review Letters* **112**, 143901 (2014).

4. Wiersma, D. S., Muzzi, A., Colocci, M. & Righini, R. Time-resolved anisotropic multiple light scattering in nematic liquid crystals. *Phys. Rev. Lett.* **83**, 4321–4324 (1999).

5. Kienle, A. Anisotropic light diffusion: an oxymoron? *Physical Review Letters* **98**, 218104 (2007).

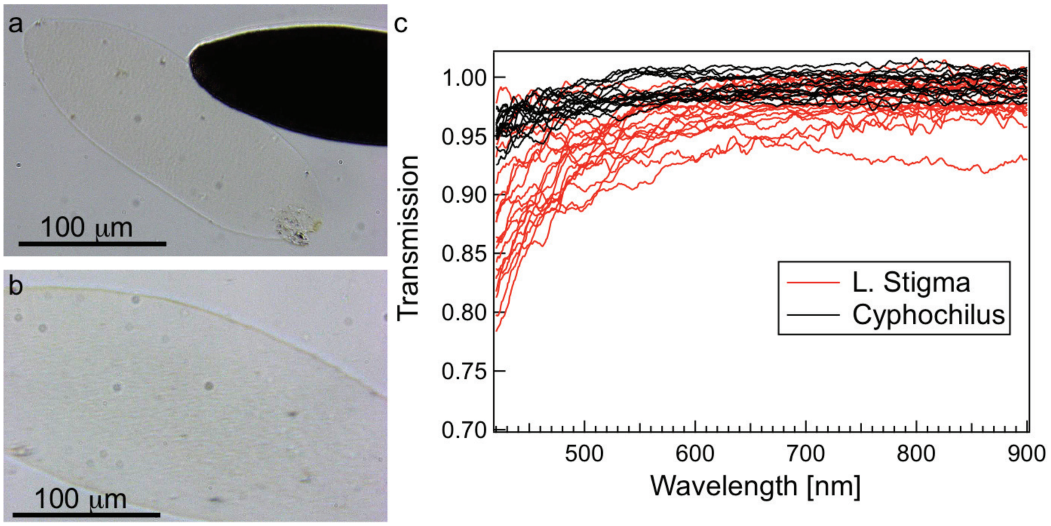

Figure 1 Refractive index match measurements. a-b Microscope images in transmission configuration of the scale of the beetles in a oil with refractive index 1.56, respectively *Cyphochilus* and *L. stigma*. The images have been taken by illuminating the sample with a condenser using a $\mathrm{NA} = 0.05$ and collecting the light with a $20\times$ objective $(\mathrm{NA} = 0.4)$. In **c** different spectra obtained from scales for the two beetle are reported.

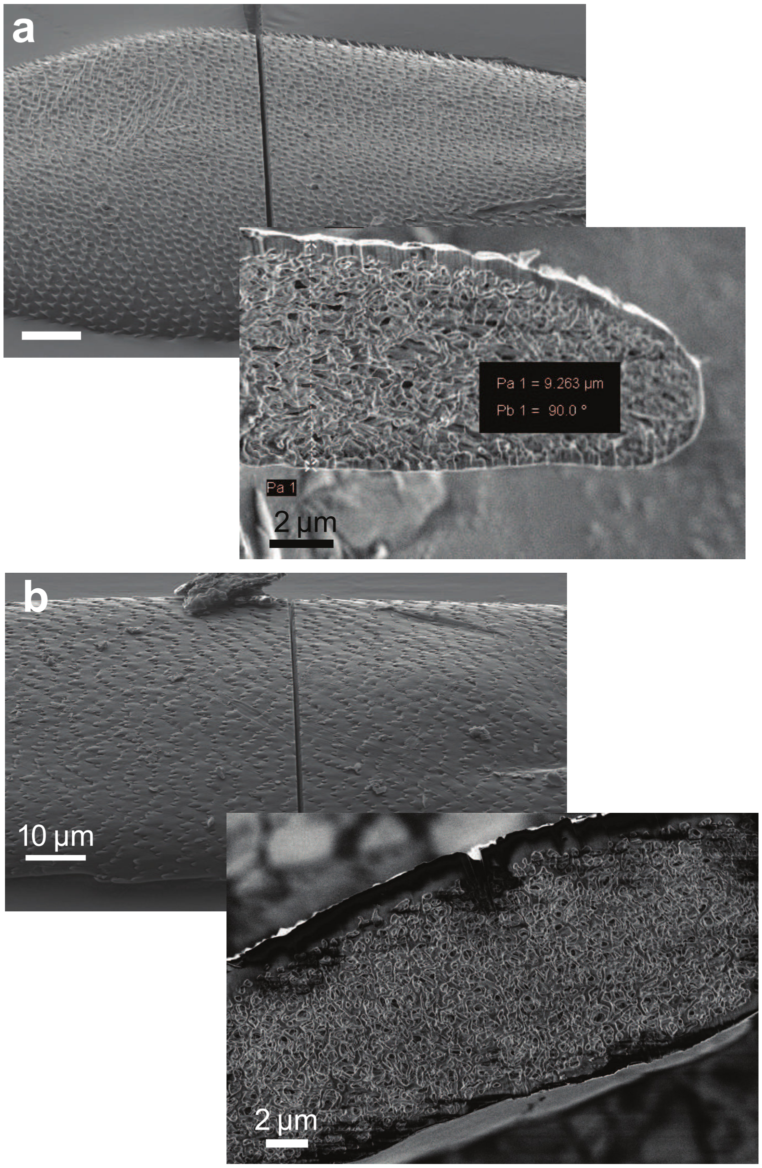

Figure 2 Cross section of the scales. a-b Scanning electron micrographs of the cross section of the scales of *Cyphochilus* and *Lepidiota Stigma*, respectively. The curvature of the scales appears evident as well as the variation of the thickness as a function of position.

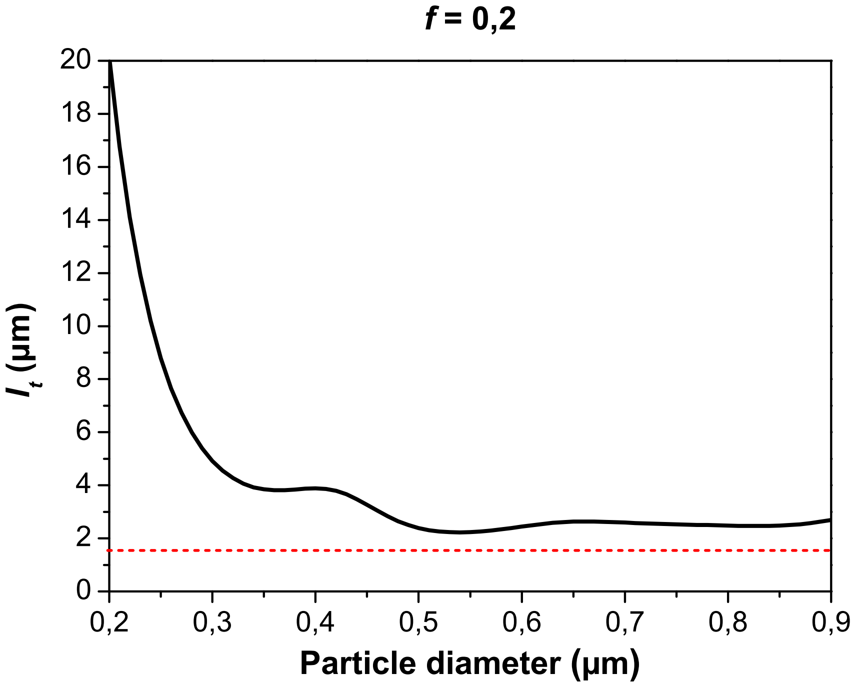

Figure 3 Transport mean free path of a particle system with $f = 0.2$. ℓ_t has been calculated by employing equation 3 as a function of the particle diameter at fixed wavelength (810 nm). The red dashed line indicates ℓ_t found for *Cyphochilus*. Clearly, the predicted ℓ_t is well above it for all particle sizes.

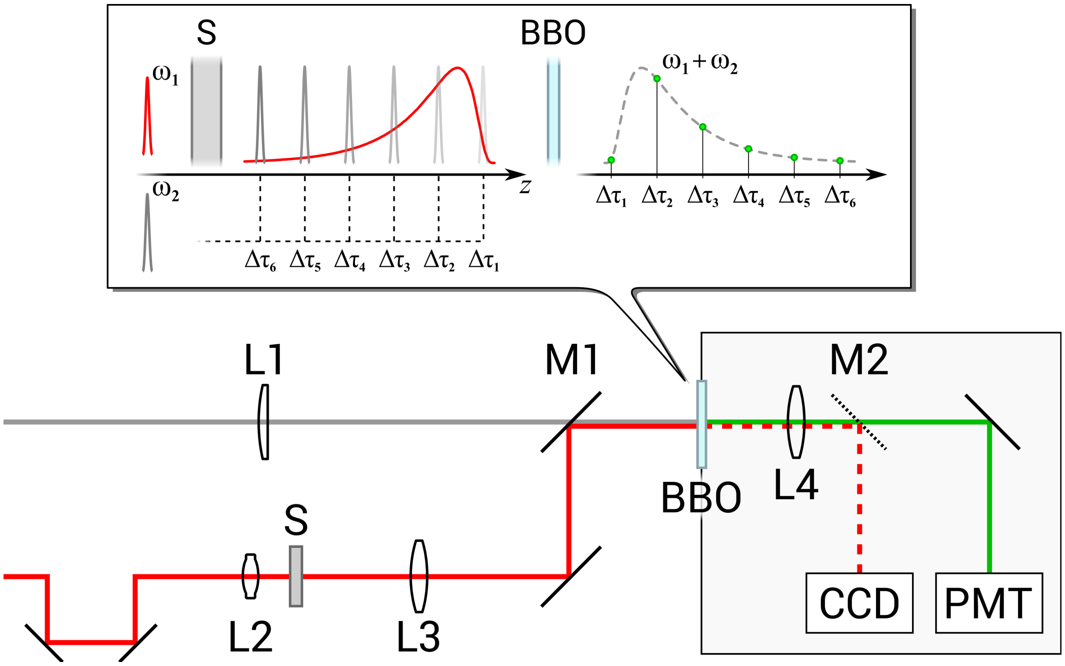

Figure 4 Optical gating setup employed for the time-resolved measurements on the white beetles scales. The inset describe the optical gating up-conversion process.
